# Multiplication of the waterborne pathogen *Cryptosporidium parvum* in an aquatic biofilm system

**DOI:** 10.1186/1756-3305-6-270

**Published:** 2013-09-19

**Authors:** Wan Koh, Peta L Clode, Paul Monis, RC Andrew Thompson

**Affiliations:** 1 School of Veterinary and Life Sciences, Murdoch University, South Street, Murdoch, WA 6150, Australia; 2 Centre for Microscopy, Characterisation and Analysis, The University of Western Australia, 35 Stirling Hwy, Crawley, WA 6009, Australia; 3 South Australian Water Corporation, 250 Victoria Square, Adelaide, SA 5000, Australia

**Keywords:** *Cryptosporidium*, Biofilms, Extracellular multiplication, Water, qPCR, Fluorescence

## Abstract

**Background:**

In natural aquatic environments biofilms are known to act as environmental reservoirs for *Cryptosporidium parvum* oocysts. However, the fate of these oocysts within biofilms has yet to be determined.

**Methods:**

This study aimed to identify if biofilms have the ability to support the multiplication of *Cryptosporidium* by measuring the change in parasite number over time using quantitative polymerase chain reaction (qPCR) and detecting the possible extracellular developmental stages using a combination of confocal microscopy and immunolabelling techniques. *Pseudomonas aeruginosa* biofilm flow cell systems were established and *C. parvum* oocysts were constantly supplied over a six day period.

**Results:**

A significant (P < 0.001) increase in *Cryptosporidium* was detected as the biofilm matured, with the total number of *C. parvum* multiplying 2–3 fold during this period. With this, various *Cryptosporidium* developmental stages (sporozoites, trophozoites, type I and II meronts) were identified from the biofilm.

**Conclusion:**

This is the first study demonstrating that biofilms not only serve as an environmental reservoir for oocysts, but are also capable of supporting the multiplication of *Cryptosporidium* over time in an aquatic environment.

## Background

*Cryptosporidium parvum* is a zoonotic waterborne pathogen found worldwide [1], with *Cryptosporidium* oocyst levels commonly monitored in urban water distribution systems [2]. If oocysts are ingested by a suitable host, which includes humans and livestock [3], the oocyst undergoes several cycles of multiplication via both asexual (sporozoites, trophozoites) and sexual processes (merozoites and microgametes) [4]. Historically, it has always been assumed that *Cryptosporidium*, like other apicomplexans, can only multiply intracellularly by forming an extracytoplasmic parasitophorous vacuole in host intestinal cells and that they are not able to multiply outside of the host [5].

There are, however, an increasing number of *in-vitro* studies, both in cell and cell-free [6-12] cultures, demonstrating that *Cryptosporidium* may not be an obligate intracellular parasite and can in fact multiply extracellularly. This capacity to multiply both intracellularly and extracellularly may reflect the fact that *Cryptosporidium* is closely related to gregarine protozoa [9,11,13], which can also multiply by either means [14]. The ability of *Cryptosporidium* to multiply extracellularly also indicates that they are able to salvage nutrients required for multiplication from their environment, despite lacking mitochondria and associated metabolic capabilities [15]. Independent of the mechanisms, it is clear that the surrounding environment plays an important role in shaping the life cycle of *Cryptosporidium*.

Biofilms are highly efficient and stable ecosystems [16] that are formed mainly by bacteria [17-19], creating a favourable micro-environment that can support the survival and growth of other micro-organisms under prolonged periods of environmental stress [16]. Biofilms have been shown to serve as an environmental reservoir for *Cryptosporidium* oocysts in aquatic environments [20-22] and may be responsible for the occurrence of sporadic *Cryptosporidium* outbreaks [23]. Therefore, there is a current need to better understand *Cryptosporidium* behaviour in biofilm environments, especially in relation to water distribution systems [18,22] and to investigate whether *Cryptosporidium* oocysts captured within biofilms can utilise this nutrient rich micro-environment to survive and multiply.

The aim of this study was to investigate whether biofilms can support the multiplication of *Cryptosporidium* in aquatic environments. Model flow cell biofilm systems were developed and confocal laser scanning microscopy coupled with image analysis was used to quantitatively compare biofilm thickness between pure biofilm cultures and biofilms exposed to *Cryptosporidium* oocysts. Quantitative PCR and a combination of confocal microscopy and immunolabelling were used to monitor *Cryptosporidium* within the biofilm system over a six day period.

## Methods

### Bacterial strains and media

Wild type *Pseudomonas aeruginosa* bacteria (PA01) were used to establish the biofilms for this study. Before incubating into the biofilm flow cell system, *Pseudomonas* cultures were maintained on *Pseudomonas* agar (Beckon-Dickson) at 37°C. A 10% solution of tryptic soy broth (Beckon-Dickson) was used as the flow-through media in all biofilm experiments.

### Parasite isolation and purification

*C. parvum* cattle genotype (Swiss cattle C26) oocysts were obtained from the Institute of Parasitology, University of Zurich and were subsequently passaged through and purified from ARC/Swiss mice as described by Meloni and Thompson [24]. Purified oocysts were stored in 1 x phosphate buffered saline (PBS) with antibiotics (10,000 U penicillin G and 0.01 g streptomycin; Sigma) at 4°C before use. Oocysts used in biofilm experiments were less than 4 weeks old and were decontaminated with 2% household bleach at room temperature. Identical batches of oocysts were used for parallel control experiments.

### Flow cell biofilm systems

**Figure 1:**
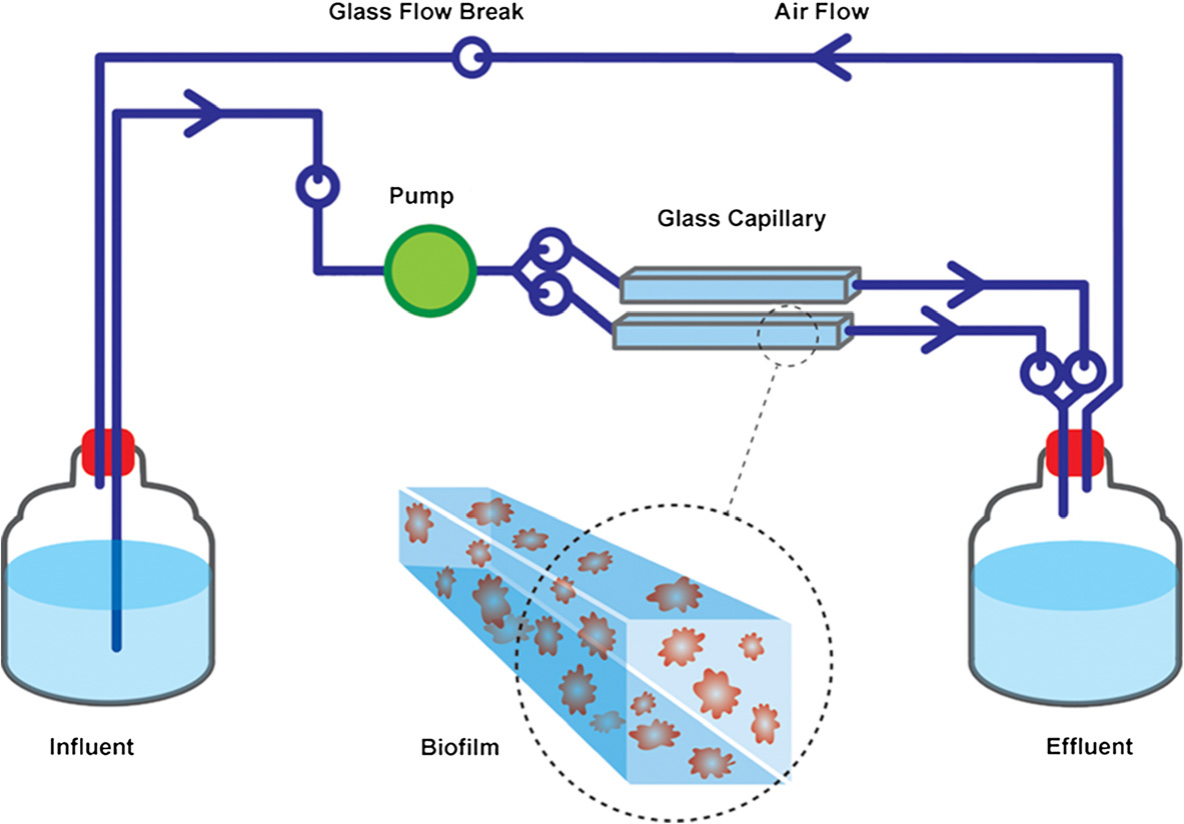
**Schematic diagram of the capillary flow cell biofilm system used in this study.** System and picture modified from Werner *et al.*[25].

Flow cell biofilm systems were set up as described by Werner *et al*. [25], except that here, the system was modified to be a fully closed system with no air intake, to prevent air contamination (Figure 1). Instead, two capillary flow cells (20 cm in length) were run in parallel, and a silicon tube was attached between the influent and effluent to ensure a smooth air flow. Hence, no bubbles were observed in the liquid flow. Five-litre glass bottles were used for both influent and effluent media. All experiments were performed at room temperature under sterile and dark environmental conditions.

*P. aeruginosa* inoculum with a turbidity equivalent to that of one McFardland standard was prepared from *Pseudomonas* agar and 1 ml of inoculum was transfered to the flow cells as described by Werner *et al.*[25]*.* To allow bacterial attachment to the surface of the flow tube, no flow was initiated for the first 24 h. Decontaminated oocysts were injected into the influent medium and the flow initiated and continued (60 ml h^-1^) for one, three or six days. Biofilms exposed to *Cryptosporidium* oocysts are hereafter referred to as *Cryptosporidium*–exposed biofilm samples.

The volume of influent medium and the number of introduced oocysts were adjusted according to the duration of the experiment (number of days). The experiment was designed such that 5 l of 10% tryptic soy broth was sufficient for a three-day experiment. The number of oocysts introduced in the influent medium was calculated so that the biofilms received 1 × 10^6^ oocysts every 24 h. In order to avoid air contamination, the oocysts were introduced at the beginning of the experiment (for all experiments), and during an influent medium change after three days (for the six day experiment). Two controls were set up simultaneously:

1) No biofilm was developed. Decontaminated oocysts were added into the flow system at similar rates, but without any biofilm established within the flow system. This sample is presented as the biofilm-free control.

2) No oocysts were added. Biofilms were grown in the flow system, without the introduction of *Cryptosporidium* oocysts. This sample is presented as the biofilm-only control.

All experiments utilised two flow tubes simultaneously for each treatment and were repeated three times.

### Biofilm thickness

Film Tracer calcein red orange (Invitrogen, Australia) was used to stain the biofilm for confocal laser scanning microscopy imaging and analysis. The biofilms were stained according to the protocol provided with the stain. Briefly, calcein red orange stain was introduced into the living biofilm within the flow tube using a peristaltic pump at a rate of 60 ml h^-1^ and incubated in the dark for 60 min at room temperature. Calcein red orange stain was similarly flushed from the system and the biofilms incubated with sterile water. Confocal images of biofilms were acquired from the flow cells directly using 576 nm and 590 nm laser excitation/emission wavelengths. Average biofilm thickness was determined from three regions - the left, right and middle area - of the flow cell. For both biofilm-only and *Cryptosporidium*-exposed biofilms, serial sections in the *xy* plane were obtained at 0.44 μm (one day old biofilm) and 2–10 μm intervals (three and six day old biofilms) along the *z*-axis, and the *z*-stack image was then analysed with COMSTAT II [26].

### DNA extraction

Following thickness measurements, biofilms were dispersed by incubation in 500 nmol l^-1^ sodium nitriporusside (Sigma Aldrich, NSW, Australia) overnight, after which the flow cells were gently washed with 1 x PBS several times to completely detach the biofilms from the flow cell surface [27]. The cell suspensions were further washed several times with 1 x PBS to remove any residual nitric oxide. Biofilms from the flow cell were resuspended in 1 x PBS to a final volume of 400 μl. To quantify the total number of parasites present at the end of each experimental period, the dispersed biofilms from the effluent were also collected and resuspended to a final volume of 2 ml.

Aliquots of flow cell biofilms (100 μl) and effluent biofilms (500 μl) were used for DNA extraction. The freeze-thaw DNA extraction method was used for all samples with the process being repeated 12 times to release DNA from *Cryptosporidium*. The resulting DNA suspension was further purified as described by the Promega Genomic DNA extraction kit protocol, except that the cell lysis step was modified from 15 min to 30 min incubation to allow complete lysing of biofilm aggregates. The purified DNA was eluted in a final volume of 50 μl in the DNA suspension buffer provided with the kit.

### Quantitative polymerase chain reaction (qPCR)

The DNA-based technique of quantitative polymerase chain reaction (qPCR) was utilised to quantify the numbers of *Cryptosporidium* within one, three and six day old biofilms. All qPCR reactions were performed on a Qiagen Rotor Gene 2 system. Standard curves were constructed using five genomic DNA triplicates extracted from a known number of oocysts and serially diluted at 1:10 dilution ratio, calibrated to correspond from 10^0^ to 10^5^ oocysts. *C. parvum* specific primers used in this study were designed to target the glyceraldehydes-3-phosphate dehydrogenase (GAPDH) gene. The qPCR reactions contained 12.5 μl of Promega GoTaq SYBR Green master mix, 2.5 mmol l^-1^ of bovine albumin serum, 10 μmol l^-1^ primers specific for *C. parvum* (forward primer 5′ATCAAGCCGTTAAGGAAGCA3′, reverse primer 5′AAATCGGTCGAGACGACATC3′) and 5 μl of DNA to a final volume of 25 μl. Thermal cycling was performed at 95°C for 2 min to activate Go-Taq polymerase then the amplification and annealing conditions involved 40 cycles of 15 s at 94°C and 1 minute at 60°C. Data was collected during the annealing step of each cycle. After amplification, a melting curve analysis was performed to determine the specificity of the PCR product. The PCR products were incubated for 15 sec at 55°C and the temperature was increased to 95°C with a ramp rate of 0.1°C sec^-1^.

As the concentration of DNA in the biofilm samples was too high, DNA dilution was performed to increase the qPCR efficacy of detecting *Cryptosporidium* in the biofilm system. Generally, the dilution factors for detecting *Cryptosporidium* were 1:100 in one and three day old *Cryptosporidium*-exposed biofilms and 1:1000 for six day old *Cryptosporidium*-exposed biofilms. Biofilm-free and six day old biofilm-only samples (negative controls) were diluted according to the respective dilution factor of the biofilm waste samples. The total number of *Cryptosporidium* present in each experiment was obtained by adding the number of parasites within the biofilm and the number of parasites within the effluent from each experiment. Biofilm-free controls were similarly analysed to provide a comparable baseline of the number of *Cryptosporidium* parasites within the system, independent of the biofilm.

### Statistical analyses

All experimental data are presented as mean ± the standard error of the mean. Analyses were performed using GraphPad Prism, version 5 (GraphPad Software, San Diego, CA). One-way ANOVA analysis of variance was performed to determine whether there were significant differences in (i) thickness between one, three and six day old biofilm only controls (ii) thickness between one, three and six day old *Cryptosporidium*-exposed biofilms, (iii) amount of *Cryptosporidium* parasites detected from one, three and six day old *Cryptosporidium*-exposed biofilms (flow cell). Two-way ANOVA analysis of variance was used to determine whether there were significant differences in (i) thickness between *Cryptosporidium*-exposed biofilms and biofilm-only controls, (ii) the total number of *Cryptosporidium* detected at the completion of experimental and biofilm-free experiments.

### Immunolabelling

As a significant increase in *Cryptosporidium* DNA was detected in six day old *Cryptosporidium*–exposed biofilms, immunolabelling with *Cryptosporidium*-specific *Sporo-a-glo* antibody was performed on this sample to examine the possible *Cryptosporidium* developmental stages present within the biofilm. This antibody has been shown to label various *Cryptosporidium* developmental stages such as sporozoites, trophozoites, type I/II meronts and type I/II merozoites [7,28,29]. An aliquot of six day old dispersed biofilm suspension (20 μl) was fixed with 2.5% paraformaldehyde in PBS for 20 min at 4°C. The cells were pelleted and incubated with 200 μl of blocking buffer (6% BSA + 10% rat serum in 1 x PBS) for 1 h at room temperature, then washed with PBS (pH 7.4, 2 times, 5 min) and incubated with polyclonal primary antibody, *Sporo-a-glo* (Waterborne Inc) (1:100 in blocking buffers) for 2 h at 37°C. The samples were then washed again with PBS (twice for 6 min) and incubated with 40 nmol l^-1^ quantum dot 655 anti-rat secondary antibody (catalogue no Q-11621MP, Invitrogen) for 2 h at room temperature. The cells were then rinsed 3 times with 1 x PBS and resuspended to a final volume of 100 μl. For comparison, unexcysted oocysts and six day old biofilm-only samples were also similarly stained.

Experimental controls included six day old *Cryptosporidium*-exposed biofilms and excysted *Cryptosporidium* oocysts labelled with (i) primary antibody only or (ii) secondary antibody only.

### Confocal microscopy

For examination of *Cryptosporidium* biofilms samples by confocal microscopy, an aliquot of the immunolabeled suspension was immobilised and attached to coverslips using 0.01% poly-L-lysine (Sigma, USA). Prior to coverslip coating, coverslips were rinsed in 100% ethanol to remove surface dirt and any contamination and air dried in a laminar hood for 20 min. Coverslips were coated with poly-L-lysine for 20 min at room temperature and were then washed with sterile water to remove excess poly-L-lysine. After being air dried in the laminar flow, the experimental sample was then transferred onto the coverslip for attachment and incubated in a humidified box for 20 min to prevent dehydration. The sample was then analysed directly by confocal microscopy (Leica SP2 inverted) using the excitation and emission wavelength of FITC at 488/525 nm. Oil immersion was used for magnifications above 40x.

### Ethical approval

The animal work in this study was reviewed and approved by the Animal Ethics Committee of Murdoch University, Australia (Permit number: R2310/10).

## Results

### Comparison of biofilm thickness: biofilm-only vs. *Cryptosporidium*-exposed biofilm

**Figure 2:**
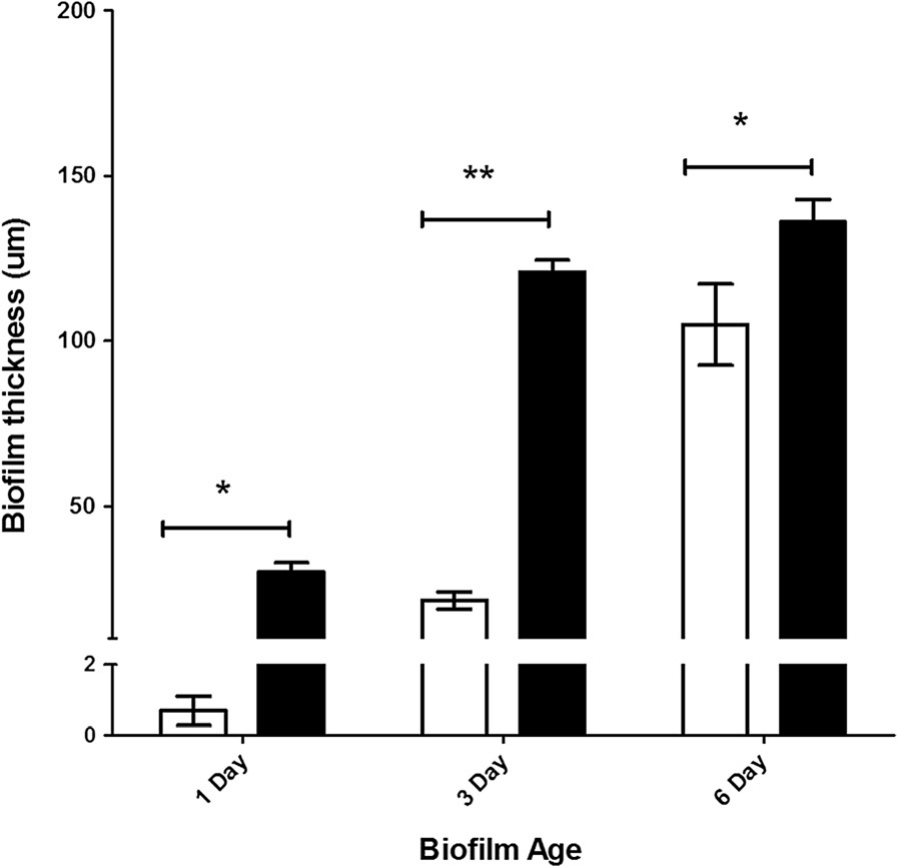
**Comparison of biofilm thickness over time between biofilm-only controls and *Cryptosporidium*-exposed biofilms.** Graph showing biofilm thickness (mean ± standard error, *n* = 6) obtained from both biofilm-only controls (white bar) and *Cryptosporidium*-exposed biofilms (black bar) using the COMSTAT II quantitative programme. Significant differences were determined using a two-way ANOVA with a post hoc test of significance (* P < 0.01; ** P < 0.0001).

Biofilm thickness in biofilm-only and *Cryptosporidium*-exposed biofilm experiments was determined using COMSTAT II [26]. For biofilm-only experiments, one day old biofilms were found to be 0.7 ± 0.4 μm thick (Figure 2), indicating that the biofilm was still developing and could therefore be described as an immature biofilm. After three days the thickness of the biofilm had increased to 21 ± 3 μm, but this was not significantly different (P > 0.05) from the one day old biofilms (Figure 2). However, a significant (P < 0.05) increase in biofilm thickness was observed in six day old biofilms (105 ± 12 μm) (Figure 2). This biofilm thickness fulfills the definition of a mature biofilm as defined by Davies *et al.*[30]*,* and hence was considered a mature biofilm.

When *Cryptosporidium* oocysts were introduced into the biofilm system, one day old *Cryptosporidium*-exposed biofilms formed immature biofilms that were 30 ± 3 μm thick (Figure 2). Unlike biofilm-only experiments, a significant (P < 0.05) increase in biofilm thickness was observed in *Cryptosporidium*-exposed biofilms after only three days (121 ± 4 μm) (Figure 2). The thickness of these three day old *Cryptosporidium*-exposed biofilms (~100 μm) could be classified as the penultimate stage of biofilm development [30,31] and was considered to be a mature biofilm. No significant (P > 0.05) increase in the thickness of this mature biofilm was observed from day three to day six in *Cryptosporidium*-exposed biofilms, with six day old biofilms found to be 136 ± 7 μm thick (Figure 2).

All *Cryptosporidium*-exposed biofilms were thicker than biofilm-only controls, but the level of significance varied. Although both biofilm-only controls and *Cryptosporidium*–exposed biofilms formed immature biofilms on day one and mature biofilms by day six, *Cryptosporidium*-exposed biofilms were always significantly (P < 0.01) thicker than the biofilm-only controls. In addition, by day three, *Cryptosporidium*-exposed biofilms had formed mature biofilms and thus had matured significantly (P < 0.0001) faster than the comparable three day old immature biofilms in the biofilm-only control.

### Association of *Cryptosporidium* with biofilms

**Figure 3:**
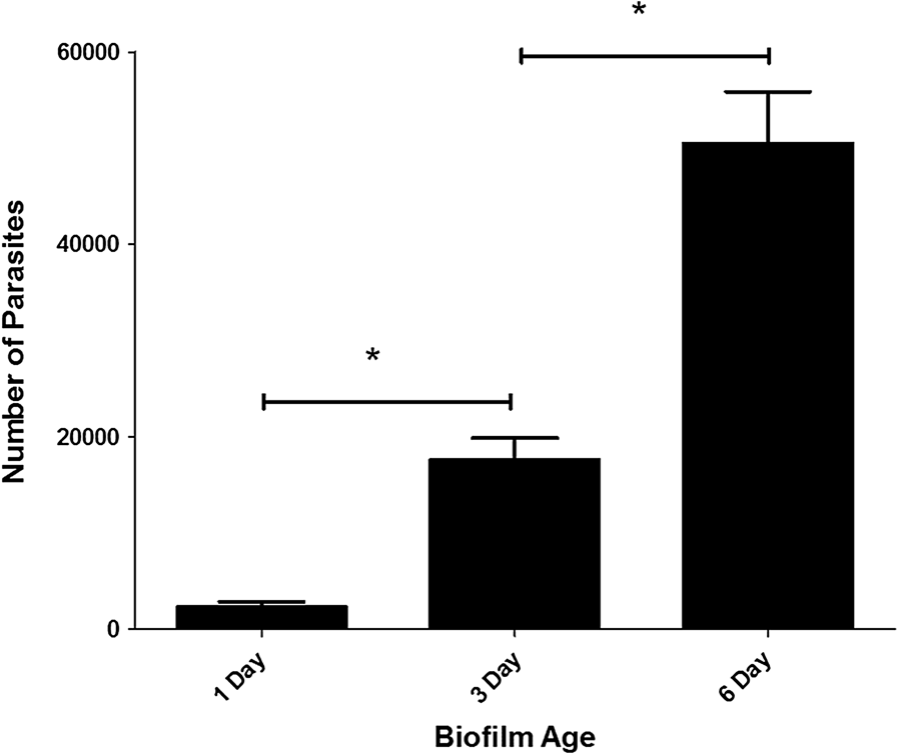
**Quantitative analysis of *Cryptosporidium *number within 1, 3 and 6 days old *Cryptosporidium*-exposed biofilms.** The number (mean ± standard error) of *Cryptosporidium* retained within *Pseudomonas* biofilms after one, three, and six days of exposure (*n* = 6 for each time period), as determined using a DNA-based qPCR approach. Significance was determined by a one-way ANOVA analysis (* P < 0.05).

Overall, qPCR revealed that the number of *Cryptosporidium* parasites retained within the biofilm increased significantly (P < 0.05) (Figure 3) and continually over time, as the biofilm increased in thickness and matured. When one and three day old *Cryptosporidium*-exposed biofilms were compared, a significant (P < 0.05) increase in both parasite number (Figure 3) and biofilm thickness (Figure 2) was observed over time. However, even when the *Cryptosporidium*-exposed biofilm had reached maturity and no longer underwent any increase in thickness (day three) (Figure 2), a significant (P < 0.05) increase in parasite numbers continued to be observed until day 6 (Figure 3).

**Figure 4:**
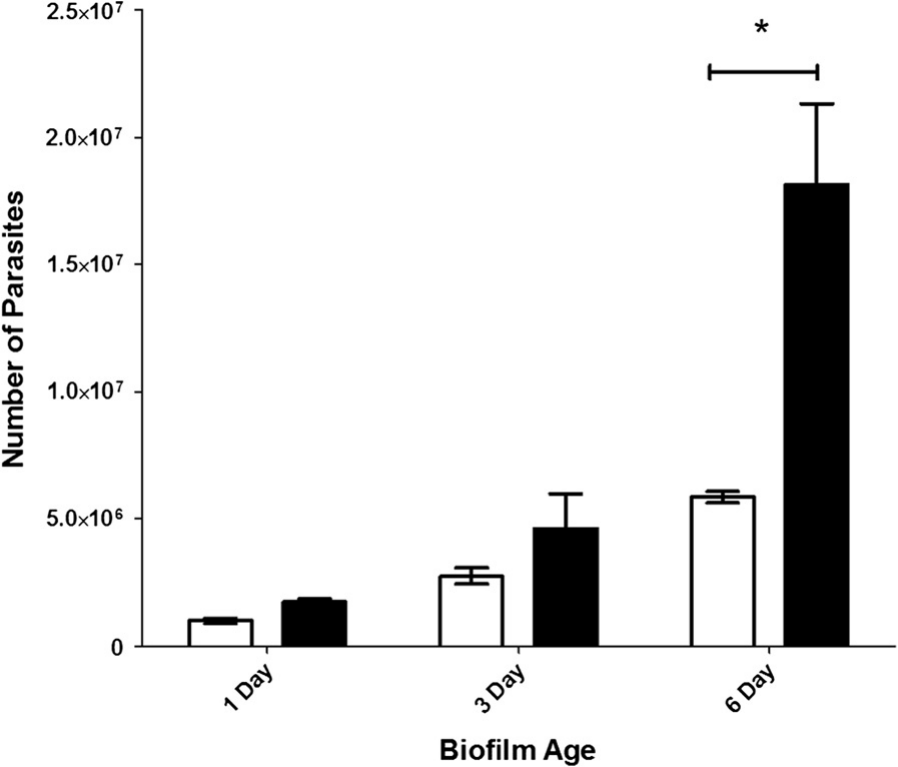
**Comparison of the total number of *Cryptosporidium *present in *Cryptosporidium-*exposed biofilms and biofilm-free control systems.** Graph showing the total number (mean ± standard error) of *Cryptosporidium* (retained in biofilm + effluent) determined at the end of each experiment (one, three and six day) for *Cryptosporidium-*exposed biofilms (black bar) (*n* = 3) and *Cryptosporidium* only (biofilm-free) control systems (white bar) (*n* = 3), as determined using a DNA-based qPCR approach. Significance was determined by a two-way ANOVA analysis with a post hoc test of significance (* P < 0.001).

Similarly, for the total number of parasites in the flow system (*i.e.* both those retained within the biofilm + those recovered from the effluent), an increase in the total number of *Cryptosporidium* was observed in both one and three day old *Cryptosporidium*-exposed biofilms, but these were not significantly (P > 0.05) different to biofilm-free controls (Figure 4). However, after six days, *Cryptosporidium-*exposed biofilms contained 2–3 fold more *Cryptosporidium* than that of the biofilm-free controls, which reflected a highly significant (P < 0.001) increase in parasite numbers when a biofilm was present (Figure 4). The significant increase in *Cryptosporidium* numbers within the biofilm flow system over the six day period implies that *Cryptosporidium* was not simply captured and accumulated but underwent multiplication within the biofilm system.

### Confocal microscopy

**Figure 5:**
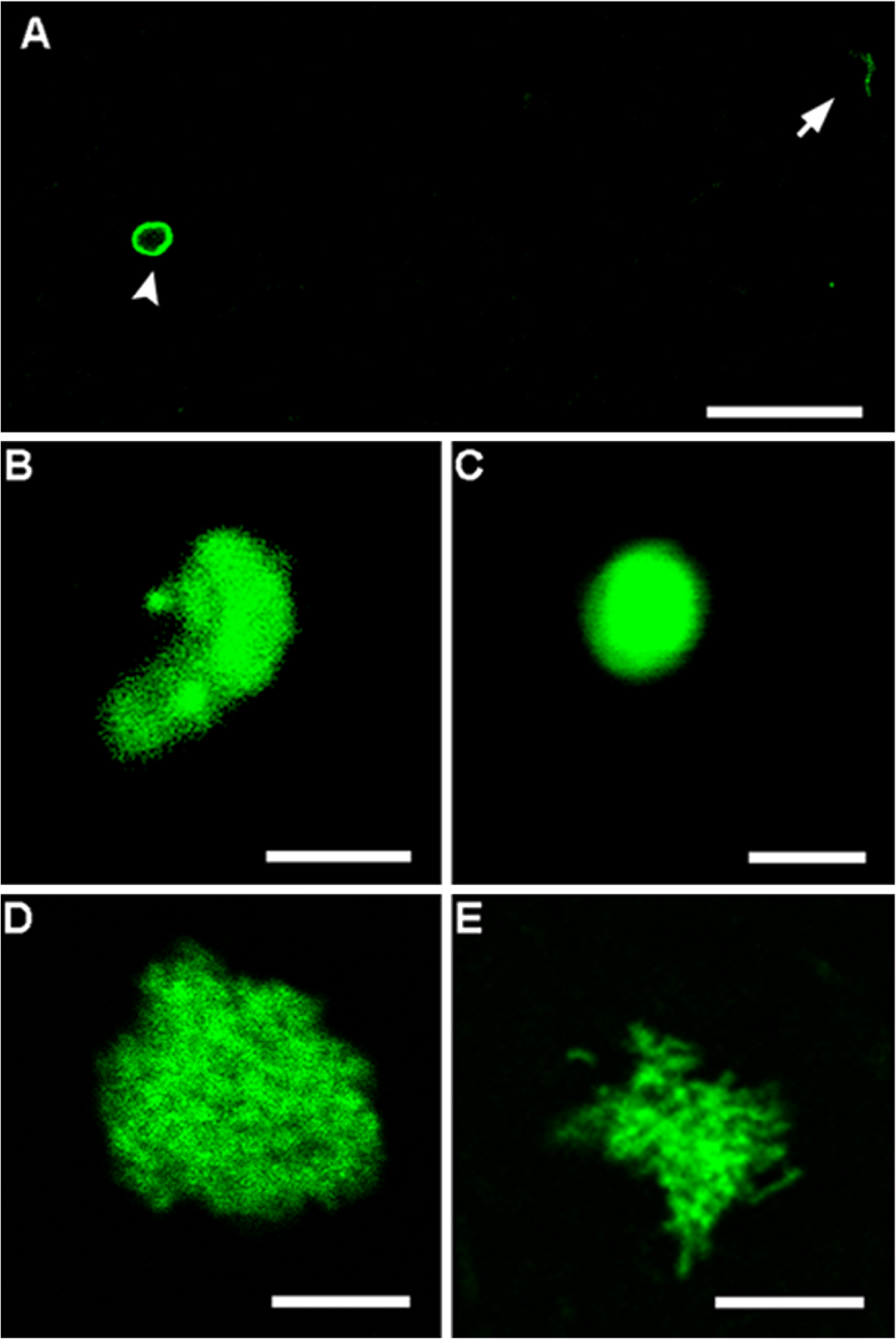
**Confocal observation of *Cryptosporidium *extracellular developmental stages identified within six day old *Cryptosporidium*-exposed biofilms. A)** excysted oocyst (indicated by arrowhead) with newly released slender sporozoite (indicated by arrow). **B)** comma shaped sporozoite; **C)** circular-shaped trophozoite; **D** and **E)** types I and II meront respectively. Scale bars: **A**: 20 μm; **B**&**C**: 2 μm; **D**&**E**: 5 μm.

The six day old *Cryptosporidium*-exposed biofilm sample (flow cell) was further analysed using confocal microscopy to examine the possible developmental stages of *Cryptosporidium* present within the biofilm. Clusters of *Cryptosporidium* and bacteria cells were frequently observed after biofilm dispersion, thus we differentiated *Cryptosporidium* from the bacterial biofilm using specific fluorescence immunolabelling of *Cryptosporidium* developmental stages. Oocyst excystation was observed to have occurred with the released of slender shaped sporozoites (Figure 5A). Comma-shaped sporozoites with a rounded posterior end and a pointed tapered anterior end were also visualised, indicating that trophozoite transformation had begun (Figure 5B). Circular shaped (2 × 2 μm) trophozoites were identified indicating completion of the trophozoite transformation process (Figure 5C). Trophozoites represent a transitional stage from sporozoites and merozoites to meronts [3]. In addition to trophozoites, two different types of meronts similar to those description by Hijjawi *et al.*[8] were also observed (Figure 5D-E). The grape-like aggregated merozoites (small with circular to oval shapes, approximately 1 × 1 μm) conform to type I meront (Figure 5D) while the aggregation of many spindle-shaped merozoites with pointed ends conform to type II meront (0.5 × 1 μm) (Figure 5E). The presence of type I/II meronts indicated that *Cryptosporidium* gametogony process had been initiated.

**Figure 6:**
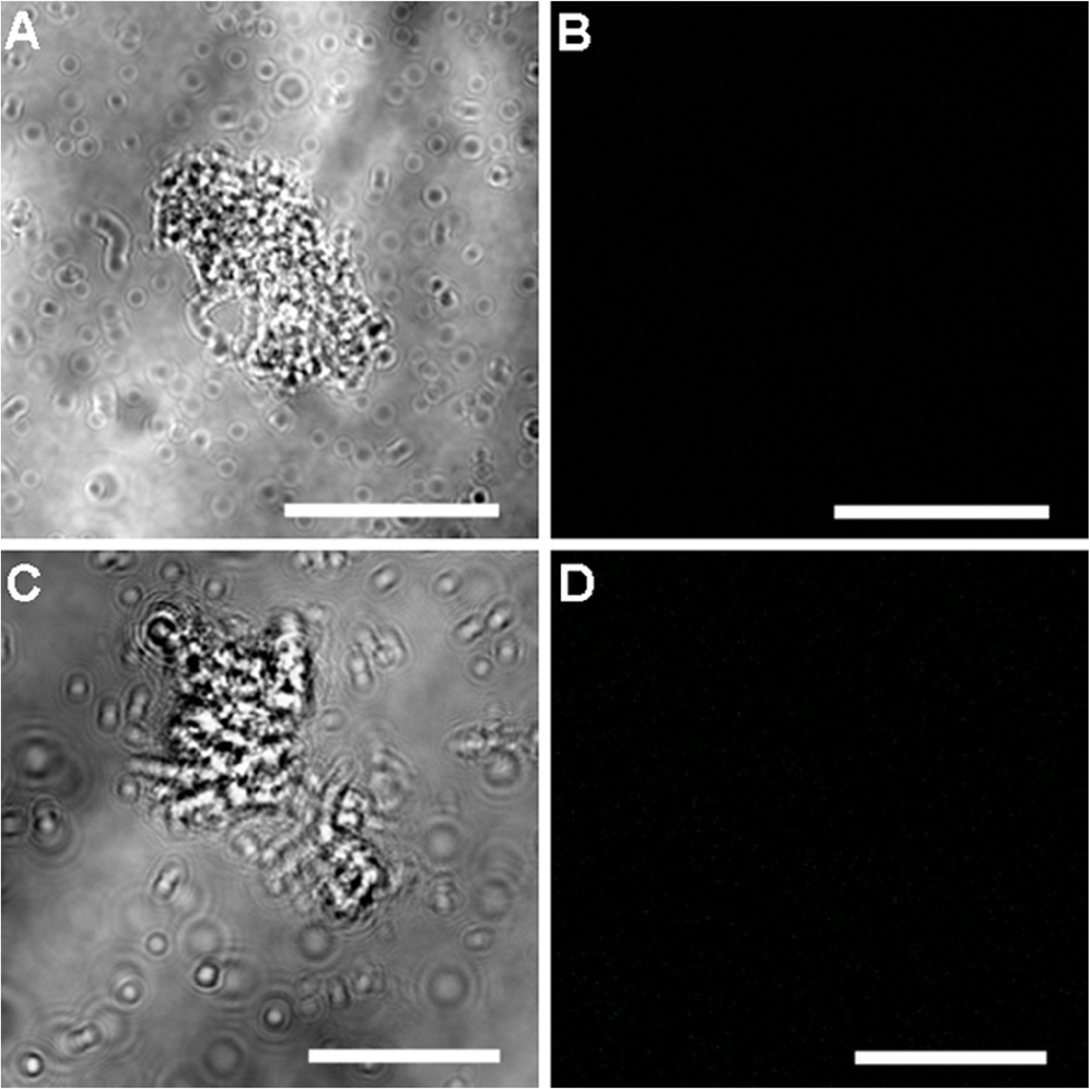
**Confocal observation of six day old *Cryptosporidium*-exposed biofilm control samples. A)** Six day old *Cryptosporidium* biofilms labelled with only primary antibody seen under transmitted light; **B)** corresponding confocal image, showing no immunolabeling. **C)** Six day old *Cryptosporidium* biofilms labelled with only secondary antibody seen under transmitted light; **D)** corresponding confocal image, showing no immunolabeling. Scale bars: **A**&**B**: 50 μm; **C**&**D**: 20 μm.

**Figure 7:**
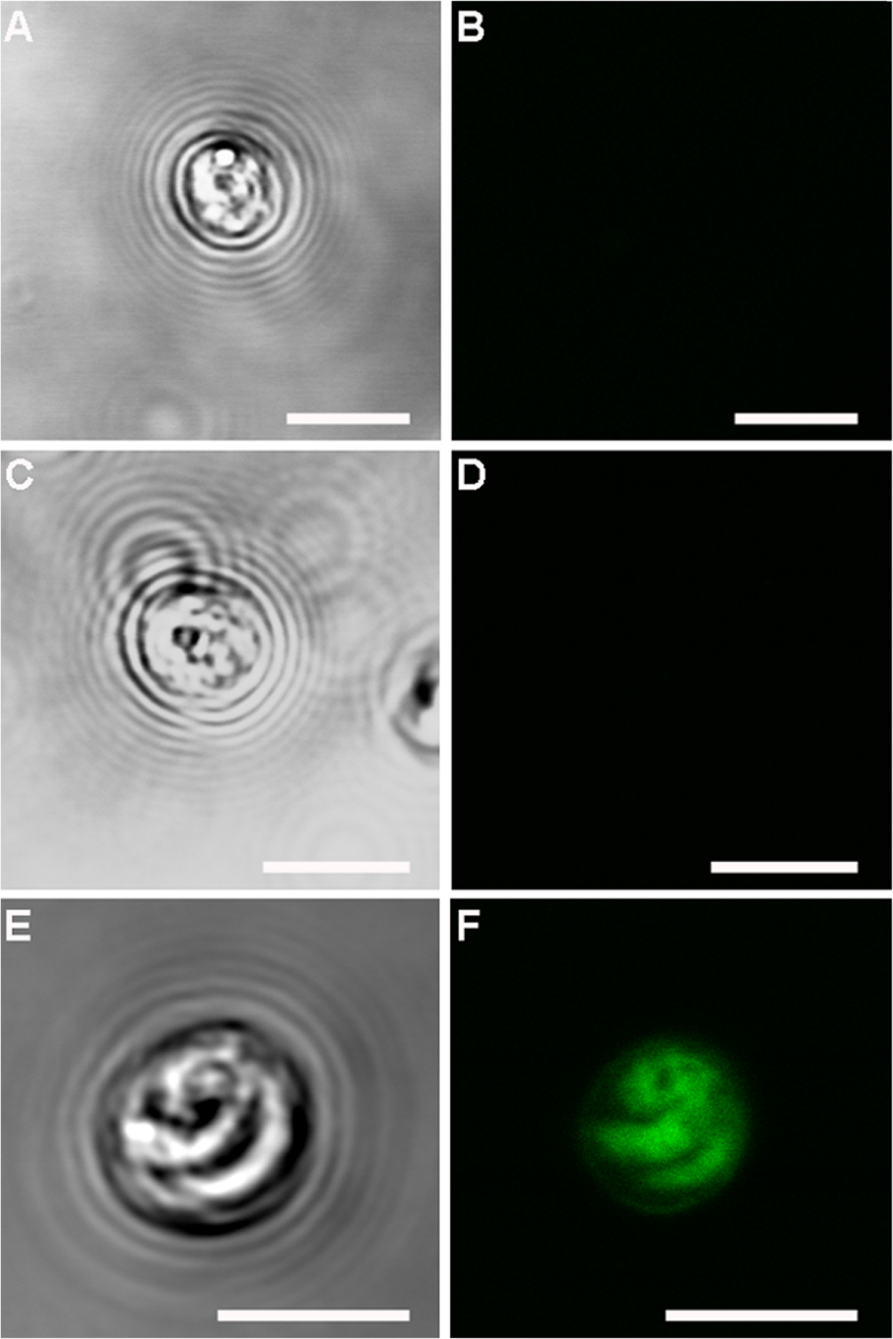
**Confocal observation of excysted oocysts control samples. A)** excysted oocyst labelled with only primary antibody seen under transmitted light; **B)** corresponding confocal image, showing no immunolabeling. **C)** excysted oocyst labelled with only secondary antibody seen under transmitted light; **D)** corresponding confocal image, showing no immunolabeling. **E)** excysted oocyst labelled with both primary and secondary antibodies seen under transmitted light; **F)** corresponding confocal image, showing oocyst intensely immunolabelled. Scale bars: 5 μm.

**Figure 8:**
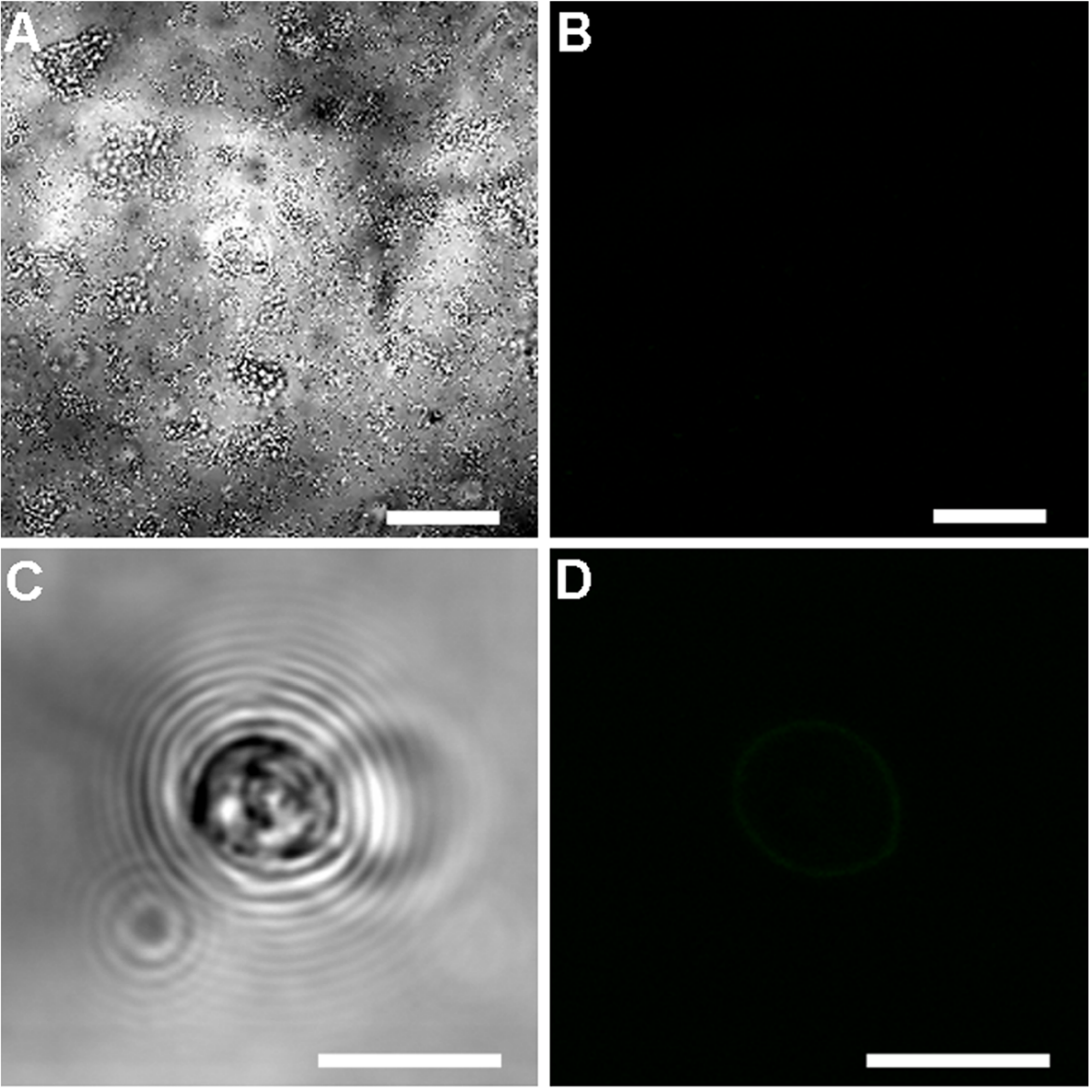
**Confocal observation of unexcysted oocyst and six day old biofilm-only control samples. A)** biofilm-only labelled with both primary and secondary antibodies seen under transmitted light; **B)** corresponding confocal image, showing no immunolabeling. **C)** Unexcysted oocyst labelled with both primary and secondary antibodies seen under transmitted light; **D)** corresponding confocal image, showing no immunolabeling Scale bars: **A**&**B**: 50 μm; **C**&**D**: 5 μm.

No fluorescent signal was observed in the control samples (six day old *Cryptosporidium*-exposed biofilms and excysted oocysts) where either the primary or the secondary antibody had been omitted (Figures 6 and 7). Fully labelled six day old biofilm-only control samples also displayed no fluorescence (Figure 8A-B). In addition, *Sporo-a-glo*, which is a *Cryptosporidium*-specific developmental stage antibody, binds only very weakly to unexcysted oocysts (Figure 8C-D). From this, we conclude that the abundant fluorescent expression observed within the biofilm samples derives from actively growing *Cryptosporidium,* rather than simply from accumulated unexcysted or degenerate oocysts. These observations are consistent with the qPCR data and show that oocysts were not simply retained within the biofilms, and had undergone the multiplication process extracellularly, as evidenced by the observation of several developmental and transitional stages.

## Discussion

This is the first study to demonstrate a significant increase in *Cryptosporidium* numbers over time within a biofilm system, highlighting that biofilms can readily provide a suitable environment for not only the retention, but also the multiplication of *Cryptosporidium* parasites, in aquatic environments. Previous studies [32,33] have shown that the number of oocysts retained within biofilms remained constant while oocysts were continually supplied to the biofilm system. However, the apparent discrepancy between these results and those presented here can be explained by the difference in the type of biofilm and methods used to detect *Cryptosporidium*. In studies by Wolyniak *et al.*[32,33], natural biofilms were used with filter sterilised creek water used as the medium. Therefore, when compared to our artificial *Pseudomonas* biofilms, their biofilms would have a different community structure and nutrient levels. In addition, through the use of the qPCR technique, our analyses quantified not only the oocysts within the system but also other *Cryptosporidium* life stages that were produced through multiplication.

The significant increase in *Cryptosporidium* DNA in six day old *Cryptosporidium* exposed biofilm was further supported by confocal microscopy observation. Cell free culture studies by Hijjawi *et al*. [7,8], Karanis *et al.*[10] and Zhang *et al*. [12] support our observation that *Cryptosporidium* can multiply extracellularly, and that encapsulation within a host cell is not essential for multiplication to occur. Due to the difficulty of identifying *Cryptosporidium* from a large background of bacteria from biofilms, not all *Cryptosporidium* stages were identified. Nevertheless, several key developmental stages representing both asexual and sexual reproduction were observed, including sporozoites, trophozoites, and types I and II meronts. The observation of morphological changes of *Cryptosporidium* sporozoites agrees with previous *in-vivo* and *in-vitro* culture observations [34-36], including sporozoites becoming oval shaped during the trophozoite transformation process. Although previous studies by Petry *et al*. [37] and Matsubayashi *et al*. [38] suggested these changes were due to aged sporozoites that could not multiply in the nutrient-limited cell free culture environment, our observation of subsequent *Cryptosporidium* development stages of the life cycle, such as types I and II meronts (based upon the developmental stage descriptions by Hijawi *et al*. [8]) within six day old biofilm provides evidence that these sporozoites were not simply aged sporozoites. The misinterpretations by these authors [37,38] have also been clearly defended and clarified by Karanis and Aldeyarbi [39]. Furthermore, the environments that liberated sporozoites are exposed to in aquatic biofilms are nutrient rich micro-environments that could allow *Cryptosporidium* to salvage their metabolite needs to fuel their high rate of growth and multiplication. This ability to multiply either intracellularly or extracellularly [6,8-12] suggests that *Cryptosporidium* i) is capable of extracting the nutrients required for growth and multiplication from the surrounding environment, ii) is not an obligate intracellular parasite, and iii) may be physiologically as well as genetically similar to the closely-related gregarines [40,41]. Further high resolution imaging [6,29,42,43] and flow cytometry-based studies [44-46] are now needed to fully characterise any additional life stages that may be produced within biofilms.

Interestingly, the presence of *Cryptosporidium* was also shown to significantly affect biofilm development and maturation. *Cryptosporidium-*exposed biofilms were found to form mature biofilms significantly faster than biofilms forming without exposure to *Cryptosporidium*. Consistent with this, Singleton *et al*. [47] also showed that biofilms that contain both prokaryotic and eukaryotic cells often formed extensive dense and thick mature biofilms. As the biofilm sloughs off after growing to a certain density, we are unable to conclusively determine if *Cryptosporidium* detected in the effluent was a result of cells sloughing off with the biofilm, or free floating cells. It is likely to be both. Although it is not possible to determine what proportion of the increase in the mature biofilm thickness was due to increases in the number of bacteria or of *Cryptosporidium*, our qPCR analyses demonstrated that even very immature biofilms were capable of capturing and accumulating *Cryptosporidium* oocysts. These may be incidentally incorporated into cell clusters during the biofilm aggregation process. Additionally, during transformation from an immature to a mature biofilm, matrix and water channels that form on the biofilm surface may have enhanced the adhesion of oocysts and also encased both parasites and bacteria, trapping those that were already retained within the biofilm [32,48]. However, while the thickness and maturation rate of the biofilm was affected by *Cryptosporidium*, the two factors were not strongly correlated, thus biofilm thickness cannot be reliably used as an indicator of the number of *Cryptosporidium* residing within the biofilm, a finding also concluded by other studies [32,33,49,50].

## Conclusion

In conclusion, this study shows that biofilms not only serve as an environmental reservoir for oocysts, but are also capable of supporting *Cryptosporidium* multiplication in an aquatic environment. The presence of biofilm on the pipes of water systems may pose a public health threat as *Cryptosporidium* residing within biofilms may increase in quantity over time, before being released into the water supply. With this, authorities should take into consideration the ability of *Cryptosporidium* to multiply within biofilms in aquatic environments when designing preventive measures to control *Cryptosporidium* contamination in water distribution systems.

## Competing interests

The authors declare that they have no competing interests.

## Authors’ contributions

WK, PLC, PM and RCAT designed the study; WK, PLC, PM and RCAT implemented the study; WK managed the data; WK, PLC and RCAT analysed and interpreted the data; WK wrote the paper. WK, PLC, and RCAT supervised the different phases of the study. All authors read, revised and approved the final manuscript.
